# The effect of eccentricity on visual motion prediction in peripheral vision

**DOI:** 10.14814/phy2.15877

**Published:** 2023-11-20

**Authors:** Riku Hirano, Kosuke Numasawa, Yusei Yoshimura, Takeshi Miyamoto, Tomohiro Kizuka, Seiji Ono

**Affiliations:** ^1^ Graduate School of Comprehensive Human Sciences University of Tsukuba Ibaraki Japan; ^2^ Graduate School of Medicine Kyoto University Kyoto Japan; ^3^ Japan Society for the Promotion of Science Tokyo Japan; ^4^ Institute of Health and Sport Sciences University of Tsukuba Ibaraki Japan

**Keywords:** peripheral vision, reaction time, time to contact, visual motion perception

## Abstract

The purpose of the current study was to clarify the effect of eccentricity on visual motion prediction using a time‐to‐contact (TTC) task. TTC indicates the predictive ability to accurately estimate the time‐to‐contact of a moving object based on visual motion perception. We also measured motion reaction time (motion RT) as an indicator of the speed of visual motion perception. The TTC task was to press a button when the moving target would arrive at the stationary goal. In the occluded condition, the target dot was occluded 500 ms before the time to contact. The motion RT task was to press a button as soon as the target moved. The visual targets were randomly presented at five different eccentricities (4°, 6°, 8°, 10°, 12°) and moved on a circular trajectory at a constant tangent velocity (8°/s) to keep the eccentricity constant. Our results showed that TTC in the occluded condition showed an earlier response as the eccentricity increased. Furthermore, the motion RT became longer as the eccentricity increased. Therefore, it is most likely that a slower speed perception in peripheral vision delays the perceived speed of motion onset and leads to an earlier response in the TTC task.

## 1 INTRODUCTION

Visual motion information from peripheral vision is important in daily life and the sports scene. Visual function is thought to decrease in peripheral vision. It has been reported that visual acuity and contrast sensitivity decrease markedly as retinal eccentricity increases (Hassan et al., 2016; Jacobs, 1979; Mandelbaum & Sloan, 1947; Rovamo & Virsu, 1979; Wright & Johnston, 1983). A number of studies have also revealed that reaction time (RT) to a light‐on stimulus is delayed in peripheral vision (Ando et al., 2001, 2002; Berlucchi et al., 1971; Osaka, 1976, 1978). The RT reflects the speed of information processing in the central nervous system (CNS). In addition, sensory and perceptual information often do not match, as actual time often differs from perceived time. Previous studies have shown that visual motion stimulus in the peripheral vision is perceived more slowly (Hassan et al., 2016; Tynan & Sekuler, 1982). Thus, peripheral vision may affect visual motion prediction based on speed perception.

In daily life or sports situations, it is often necessary to predict the arrival time of moving objects. For example, by predicting the speed and distance of surrounding pedestrians, bicycles, cars, etc. while walking, it is possible to maintain a safe distance. A time‐to‐contact (TTC) task has been widely used to estimate the predictive ability of the time to contact a moving object by speed perception. In this task, the participant is asked to predict the time when a moving target would arrive at a goal point. When the target disappears before the goal, the arrival time is predicted from the target speed in the visible segment (Battaglini et al., 2013). Previous studies on TTC have examined the effects of factors such as age (Benguigui et al., 2004, 2008), occlusion time (Benguigui et al., 2004), eye movement (Bennett et al., 2010), and attention (Baurès, Balestra, et al., 2018; Baurès, Maquestiaux, et al., 2018). The ability to estimate TTC improves significantly from 7 to 10 years of age (Benguigui et al., 2004), and the absolute error increases with increasing occlusion time in the TTC tasks (Benguigui et al., 2008). Although the TTC task uses target motion stimuli moving in vertical, horizontal, and depth linear motion (Bennett et al., 2010; Daneshi et al., 2020; Miyamoto et al., 2021; Peterken et al., 1991), it is uncertain the effects of eccentricity on the TTC. Previous studies have reported that TTC estimation is affected by the speed of the object, and there is a tendency for higher speed to delay the response (Bennett et al., 2010; Miyamoto et al., 2021; Peterken et al., 1991). While various factors have been investigated for the TTC, there are no studies that have investigated the influence of eccentricity. The purpose of this study was to verify the effect of eccentricity on motion prediction based on speed perception in peripheral vision. We hypothesized that as visual eccentricity increases, visual motion prediction based on the TTC task would be affected. In this study, we attempted to clarify the effect of eccentricity on temporal prediction based on speed perception.

The characteristics of motion information processing have been discussed in terms of the neuronal activity in the middle temporal (MT) and middle superior temporal (MST) areas in the posterior bank of the superior temporal sulcus (STS). In fact, it is known that regions such as V1, MT, V5, and the parietal cortex are activated as neural bases during TTC task and motion prediction (Assad & Maunsell, 1995; Bosco et al., 2008; Coull et al., 2008; Dessing et al., 2013; Monache et al., 2017; Schenk et al., 2005). In particular, V1, MT, and V5 are located in the early stages of the processing pathway of TTC perception and are thought to be involved in retaining target information during occlusion in the parietal cortex. Then, based on the information of the visual and parietal cortex, movements are planned and executed in the motor cortex. Visual information processing is distinguished into the ventral and dorsal pathways (Goodale & Milner, 1992). The former identifies the quality of the object (e.g., color and shape), while the latter identifies the visual motion (e.g., direction and speed). Visual information first travels from photoreceptor cells in the retina through magnocellular (M) cells and parvocellular (P) cells in the lateral geniculate nucleus (LGN) to the visual cortex. M cells have low spatial resolution but high temporal resolution, while P cells have low temporal resolution but high spatial resolution. M cells are the origin of the dorsal pathway, and P cells are the origin of the ventral pathway. M and P cells are projected from rod and cone cells in the retina, respectively. As eccentricity increases, the density of M cells gradually decreases, and the density of cone cells substantially decreases. Thus, the early stages of visual information processing are affected by retinal eccentricity, which in turn affects the subsequent specific processing. The MT and MST areas contain direction and speed selective cells and show increased firing rates in response to preferences (Churchland et al., 2007; Erickson & Thier, 1992; Ilg, 2008; Krekelberg & van Wezel, 2013; Maunsell & Van Essen, 1983). In fact, the MT/MST is involved in motion perception (Larcombe et al., 2018). For example, Newsome and Pare (1988) have reported that impairment of the MT in monkeys elevates motion thresholds in a direction discrimination task (Newsome & Pare, 1988). In addition, it has been reported that lesions in the MT cause impairments in speed perception (Orban et al., 1995), and microstimulation of the MT leads to a bias of the speed judgment toward preferred speed (Liu & Newsome, 2005). These studies indicate that the activity of the MT/MST neurons plays a prominent role in visual motion responses. Previous studies have shown a significant correlation between the motion reaction times (motion RT) and initiation of pursuit eye movement (Miyamoto et al., 2020; Ono et al., 2019). It is known that smooth pursuit is induced by the slip of the retinal image, and the pursuit initiation is associated with activity in the MT and MST areas (Dursteler & Wurtz, 1988; Komatsu & Wurtz, 1988; Newsome et al., 1988; Ono & Mustari, 2016). Therefore, motion RT may indirectly reflect the activities of MT and MST areas in visual motion processing. In this study, motion RT was used as an indicator of the MT and MST areas and verified the relationship with TTC. In contrast, the RT for target appearance without motion (light‐on RT) was used as an indirect processing process in the visual cortex that is non‐specific to motion perception. From these two RTs, the effect of eccentricity on the response of visual motion processing in the visual cortex was examined.

The purpose of this study was to verify the effect of eccentricity on motion prediction based on speed perception in peripheral vision. In order to perform accurate TTC, accurate visual motion perception is necessary. Therefore, TTC and motion RT would be similarly affected by eccentricity.

## 2 MATERIALS AND METHODS

### 2.1 Participants

In the TTC task, 20 graduate or undergraduate students (7 women and 13 men, mean age: 22.2 [range: 21–26] years old) participated, of which 19 (mean age: 22.1 [range: 21–26] years old) continued to participate in the motion and light‐on RT tasks. They had normal or corrected‐to‐normal vision. To determine the sample size, a‐priori Power analysis by g‐power software (Faul et al., 2007) was conducted, and the number of participants was determined based on the results of this power test. This study was conducted in accordance with the Declaration of Helsinki, and all protocols were approved by the Research Ethics Committee at the Faculty of Health and Sport Sciences, University of Tsukuba. All participants gave written informed consent.

### 2.2 Stimulus and procedure

Visual stimuli were generated using Psychophysics Toolbox extensions on Matlab (MathWorks, Natick, MA, USA), and they were shown on a CRT monitor (22‐inch, RDF223G, Mitsubishi Electric Co., Tokyo, Japan, refresh rate: 60 Hz, spatial resolution: 800 × 600 pixels, background mean luminance 60 cd/m^2^). During the experiment, the participants were seated 57 cm from the monitor and stabilized their heads by a chin rest and a forehead restraint in a dimmed room. A cylindrical handheld pushbutton was used for the responses in this experiment, and participants gripped it with their dominant hand and pressed the button with their thumb. Participants had a binocular vision of the monitor during the experiment.

### 2.3 Time to contact (TTC) task

**FIGURE 1:**
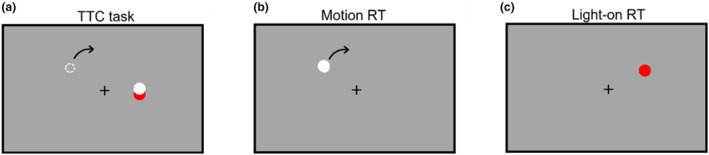
The event of each task. (a) For the TTC task, the participants react by button pressing when the target reaches the goal point. (b) For the motion RT task, the participants react by pressing the button as fast as possible when the target moved. (c) For the light‐on RT task, the participants react by pressing the button as fast as possible when the target appeared.

The TTC task was to press the button at the time when the target dot would arrive at a goal dot point (Figure 1a). Participants were asked to fixate on a fixation point of the center of the display throughout the experiment. The target consisted of a white dot (diameter of 0.5°) presented on a uniform gray background. After the 1200–1800 ms fixation point displayed, the target moved on a circular trajectory at a constant tangent velocity (8°/s) in 2000–3000 ms. There were five target trajectory eccentricities (4°, 6°, 8°, 10°, 12°), randomized within the block. The target dot always moved clockwise orientation. Consequently, the target moved with constant eccentricity. Furthermore, the goal was presented as a red dot (diameter of 0.5°) on the target trajectory. In addition, we set visible and occluded conditions. In the visible condition, the target dot was presented throughout the experiment. In the occluded condition, the target dot was occluded 500 ms before the target arrival time to the goal. Each set consisted of 30 trials, and a total of five blocks were conducted. Consequently, in total, 3000 trials (30 trials × 5 blocks × 20 participants) were performed. The eccentricity condition was randomized within the trial.

### 2.4 Motion reaction time task

The motion RT task was to press the button as fast as possible when a target dot moved (Figure 1b). Participants were required to fixate to a fixation point presented in the center of the monitor, and this task included five eccentricity conditions (4°, 6°, 8°, 10°, 12°). After the 1200–1800 ms fixation point displayed, the target consisted of a white dot moving on a circular trajectory at a constant tangent velocity (8°/s). The target always moved in a clockwise direction, and its starting position was randomized. One set consisted of 40 trials, and two blocks were conducted. Consequently, in total, 1520 trials (40 trials × 2 blocks × 19 participants) were performed. The eccentricity condition was randomized within the block.

### 2.5 Light‐on reaction time task

The light‐on RT task was similar to the motion RT task. Participants were to press the button as fast as possible when the target appeared (Figure 1c). During the task, participants fixated on a fixation point throughout the experiment. After the 1000–2000 ms fixation point was displayed, the target appeared. In this study, we presented a target at one of the five eccentricity positions (4°, 6°, 8°, 10°, 12°). Red target dots (diameter of 0.5°) were presented randomly. One set consisted of 40 trials, and 2 blocks were conducted. Consequently, in total, 1520 trials (40 trials × 2 blocks × 19 participants) were performed. The eccentricity condition was randomized within the trial.

### 2.6 Data collection and analysis

Eye movements from the right eye were detected using a video‐based eye tracking system. In this system, eye position signals were detected using an infrared camera (GS3‐U3‐41C6NIR, FLIR systems Inc., OR., USA), which captured reflections of the infrared light on the cornea and a black image of the pupil. The eye position signal was also digitized at 1 kHz with 16‐bit precision using CED‐Micro 1401 hardware (Cambridge Electronic Designs, Cambridge, UK). Prior to tasks in this study, the eye position signals were calibrated by requiring the participants to keep fixated on five target spots (diameter of 0.3°) on a uniform black background in a binocular viewing condition.

In the TTC task, we calculated the constant error (CE) by measuring the time difference between the target dot reaching the goal point and the participant's response when pressing a button. CE allows us to assess response bias, where positive values indicate overshooting and negative values indicate undershooting. We also calculated variable error (VE), which corresponds to the standard deviation and implies the variation of the TTC error. In addition, absolute error (AE) was calculated, which corresponds to the absolute value of CE. The motion RT was calculated by the time between motion onset and the button press and the light‐on RT was calculated by the time between the target appearance and the button press. During these tasks, we monitored the eye position and if eye position shifts more than 2° from the fixation point, the trial was excluded from the analysis. In addition, if the measured reaction time was greater than ±3 SD of the mean reaction time of each eccentricity, it was excluded from the analysis. In each RT task, 1520 trials were performed overall. In the TTC task, 3000 trials were performed overall. A total of 6040 trials (light‐on RT: 1520 + motion RT: 1520 + TTC task: 3000) were recorded, of which 279 trials were removed. In addition, in the RT task, the average response time of one participant was higher than the mean value of ±2 SD in the group and was excluded from the analysis, so the data of 18 participants were analyzed. These analyses were conducted by Matlab (MathWorks, Natick, MA, USA) and Excel (Microsoft, Redmond, WA, USA).

### 2.7 Statistical analysis

To verify the effect of eccentricity in the TTC task in each condition, CE and VE carried out a two‐way analysis of variance with repeated measures in two conditions (visible and occluded conditions) and five eccentricities (4°, 6°, 8°, 10°, and 12°) as within factors. A post hoc multiple comparison test was conducted with the Bonferroni correction. If the Maunchly's sphericity test was significant, the degrees of freedom were adjusted using the Greenhouse–Geisser correction. Effect sizes of ANOVA were reported as partial *η* ^2^. In addition, in order to verify the influence of eccentricity on the TTC task considering random intercepts between subjects, a two‐way analysis of variance model with fixed effects as the two conditions (visible and occluded conditions), five eccentricities (4°, 6°, 8°, 10°, and 12°), and random effects as the unique differences of individual subjects were analyzed using a linear mixed model (LMM). To verify the effects of target characteristics and eccentricity on reaction time as visual information processing time, the light‐on and motion RTs carried out a one‐way analysis of variance with repeated measures in five eccentricities (4°, 6°, 8°, 10°, and 12°) as within‐participant factors, respectively. In order to verify the difference between motion and light‐on RTs, the analysis was performed using a paired t‐test, and the effect size was reported as Cohens'*d*. All statistical tests were conducted with a significance level of 0.05 and were carried out using IBM SPSS software version 28 (SPSS inc., IL., US). LMM analysis was performed using JASP software version 0.18.

## 3 RESULTS

### 3.1 Time to contact task

**FIGURE 2:**
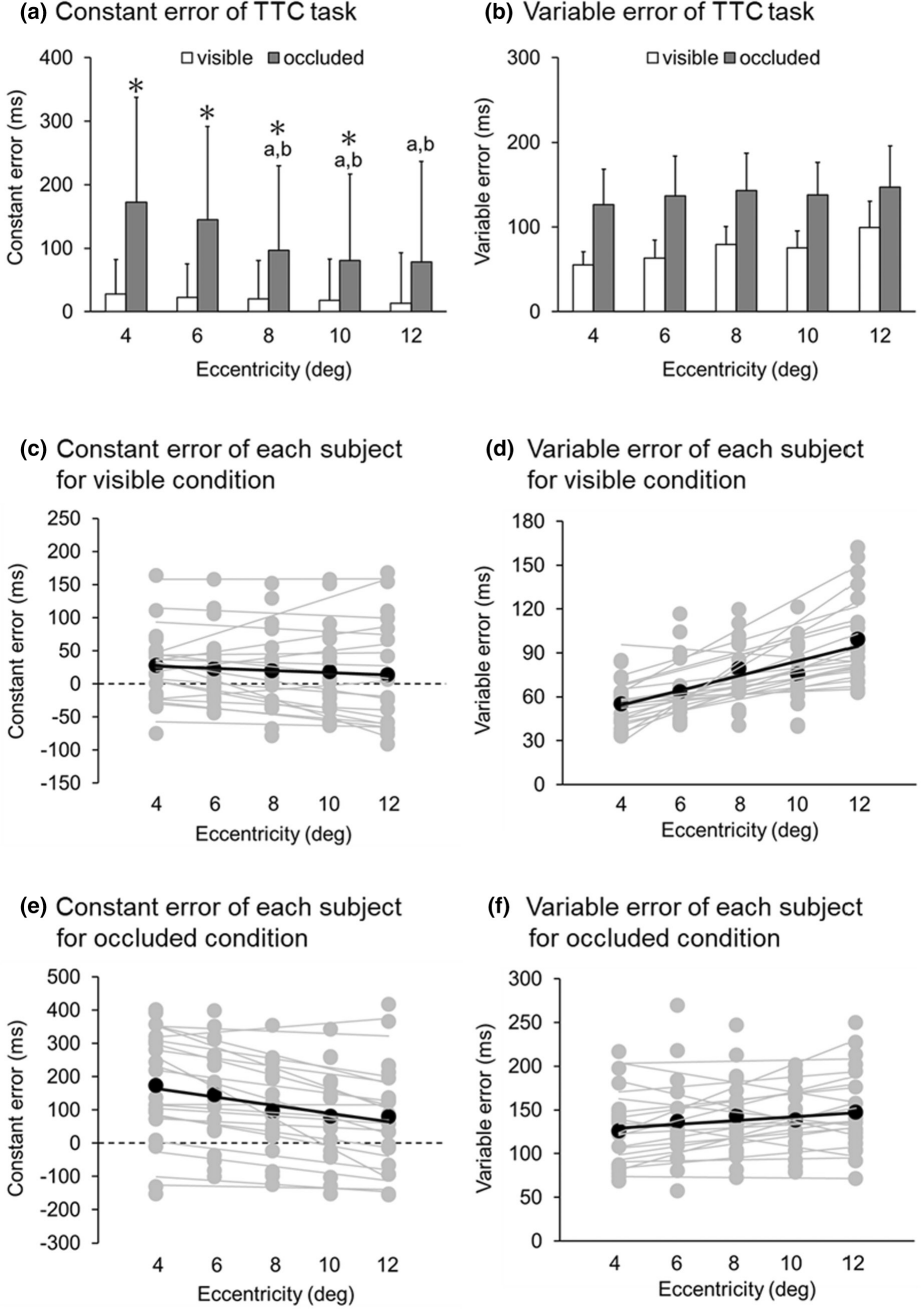
(a) The mean and standard deviation of the constant error (CE) of the time‐to‐contact (TTC) in visible and occluded conditions for each eccentricity (4°, 6°, 8°, 10°, and 12°). (b) The mean and standard deviation of the variable error (VE) of the TTC in visible and occluded conditions for each eccentricity (4°, 6°, 8°, 10°, and 12°). The white and gray bars represent the mean values of the visible and occluded conditions, respectively. The black vertical lines represent the standard deviation of the visible and occluded conditions. Positive values of CE indicate delayed responses to the TTC, while negative values indicate early responses. *: a significant difference between visible and occluded conditions. a: a significant difference versus 4°. b: a significant difference versus 6°. (c) The mean values of CE of each participant in the visible condition for each eccentricity. The gray and black symbols represent the mean and individual values for each participant, respectively. The gray and black lines represent the mean and individual regression lines for each participant, respectively. (d) The mean values of VE of each participant in the visible condition for each eccentricity. The gray and black symbols represent the same as C. (e) The mean values of CE of each participant in the occluded condition for each eccentricity. The gray and black symbols represent the same as C. (f) The mean values of VE of each participant in the occluded condition for each eccentricity. The gray and black symbols represent the same as C.

**FIGURE 3:**
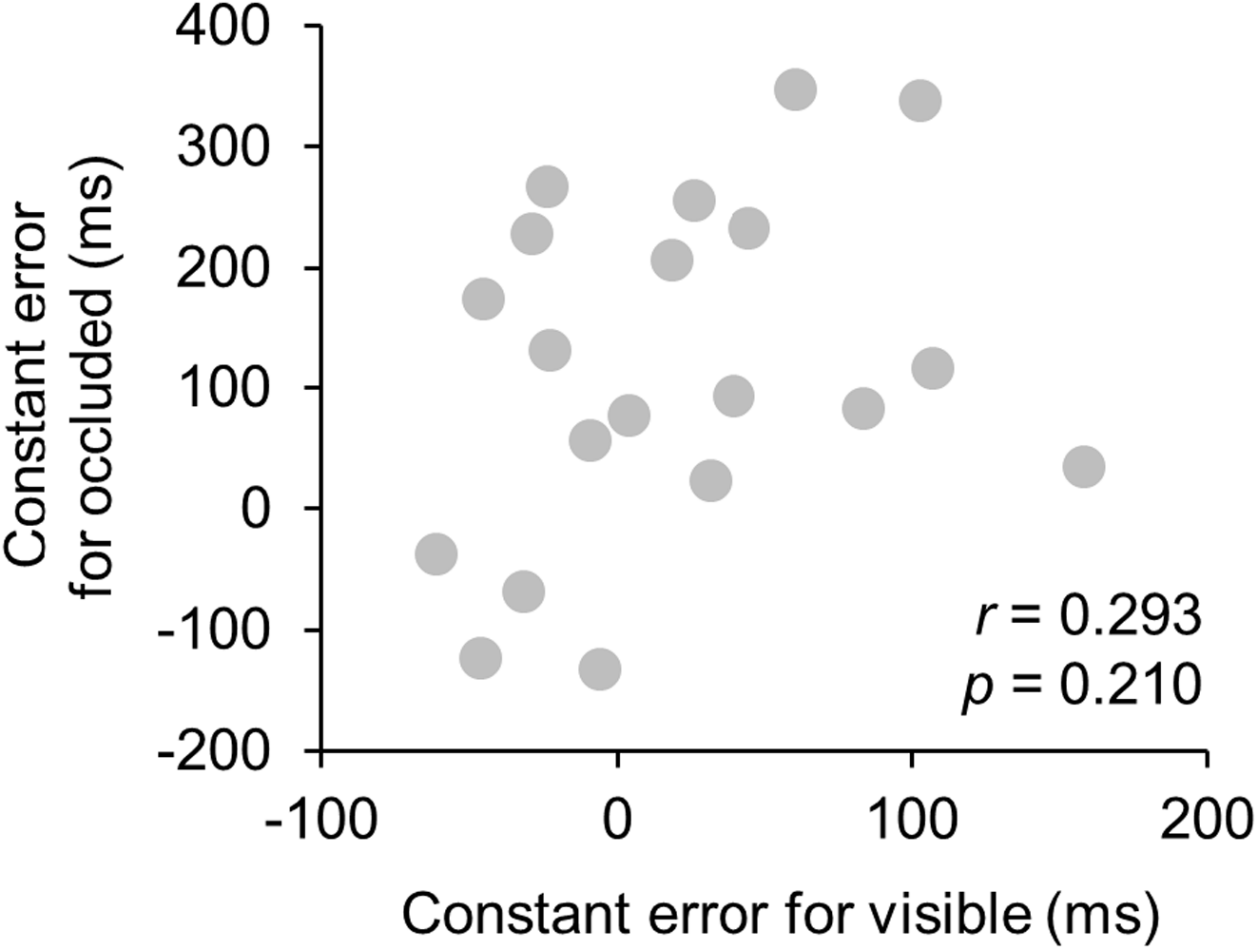
The relationship between the CE for the visible and occluded conditions. Each dot shows the mean value for overall eccentricity in each participant.

Figure 2 shows the mean values (± SD) and each participant value of CE of the TTC task for each condition and eccentricity. Table 1 shows the mean and standard deviation of CE of the TTC task in the visible and occluded conditions. The two‐way analysis of variance for CE showed a significant interaction between condition and eccentricity (*F* (2.740, 52.067) = 12.824, *p* < 0.001, Partial *η* ^*2*^ = 0.403). There were significant main effects of condition and eccentricity on CE (*F* (1.000, 19.000) = 9.517, *p* < 0.001, Partial *η* ^2^ = 0.334, *F* (1.908, 36.259) = 10.988, *p* < 0.001, Partial *η* ^2^ = 0.366, respectively). Post hoc multiple comparisons showed that CE in the occluded condition was significantly larger than in the visible condition for eccentricities of 4°, 6°, 8° and 10° (*p* < 0.001, *p* < 0.05, *p* < 0.05, *p* < 0.05, respectively), indicating that the participants show a delayed response in the occluded condition. Regarding eccentricity, in the visible condition, there was no significant difference in CE with increasing eccentricity. However, when considering the occluded condition, subsequent post hoc multiple comparisons indicated a significantly larger CE at the eccentricity of 4° compared to 8°, 10°, and 12° (*p* < 0.05 for all comparisons with the eccentricities of 8°, 10°, and 12°). Furthermore, CE at the eccentricity of 6° was significantly larger than that at 8°, 10°, and 12° (*p* < 0.05, *p* < 0.001, *p* < 0.001, for the eccentricities of 8°, 10°, and 12°). Moreover, a two‐way analysis of variance for VE showed no significant interaction between condition and eccentricity (*F* (4.000, 76.000) = 1.764, *p* = 0.145, Partial *η* ^*2*^ = 0.085). However, there were significant main effects of condition and eccentricity on VE (*F* (1.000, 19.000) = 48.868, *p* < 0.001, Partial *η* ^*2*^ = 0.720, *F* (4.000, 76.000) = 7.252, *p* < 0.001, Partial *η* ^*2*^ = 0.276, respectively). Furthermore, as a result of the analysis of the linear mixed model, significant main effects were observed on condition (*F* = 9.513, *p* < 0.05) and eccentricity (*F* = 6.124, *p* < 0.001), and significant interactions were also observed (*F* = 15.908, *p* < 0.001). Figure 3 shows the correlation between the mean CE for the visible and occluded conditions of each participant. There was no significant correlation between CE for the visible and occluded conditions (*r* = 0.293, *p* = 0.210, *R* ^2^ = 0.085).

**TABLE 1:** Mean measures with a standard deviation of the mean.

	4°	6°	8°	10°	12°
CE in visible (ms)	27.78 (± 54.70)	22.40 (± 53.04)	19.83 (± 60.55)	17.79 (± 65.05)	13.55 (± 79.33)
CE in occluded (ms)	172.72 (± 164.80)	144.49 (± 147.57)	96.31 (± 133.10)	80.38 (± 136.54)	78.64 (± 157.76)
Motion RT (ms)	285.36 (± 21.50)	290.89 (± 20.50)	296.73 (± 24.18)	297.73 (± 28.23)	298.76 (± 27.19)
Light‐on RT (ms)	255.38 (± 14.59)	256.77 (± 21.67)	259.78 (± 20.56)	264.46 (± 25.06)	270.08 (± 23.56)

### 3.2 Motion reaction time and light‐on reaction time task

**FIGURE 4:**
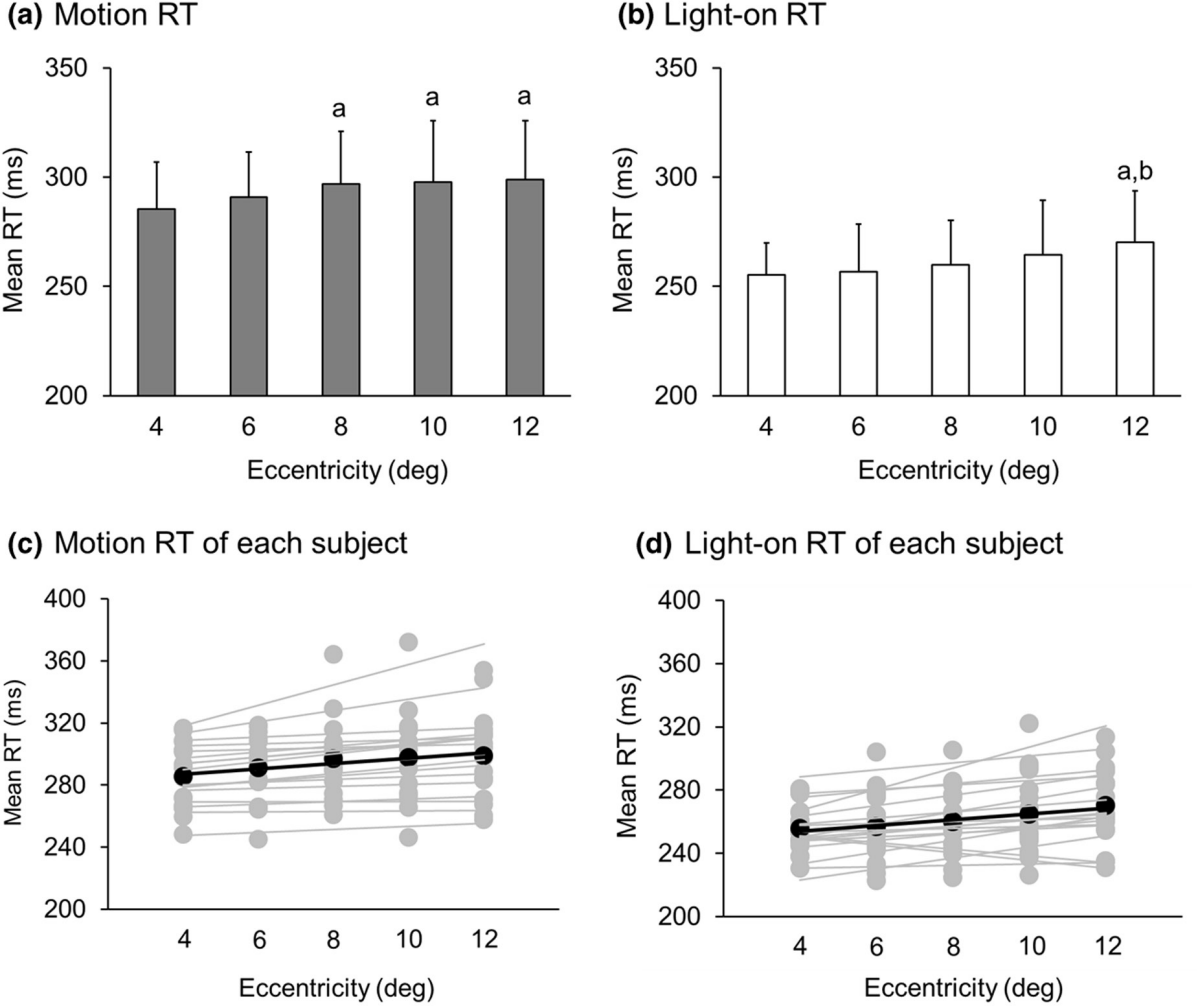
(a) The mean and standard deviation of motion RT for each eccentricity. (b) The mean and standard deviation of light‐on RT for each eccentricity. The bars and vertical lines show the mean and standard deviation. a: a significant difference versus 4°. b: a significant difference versus 6°. (c) The mean values of motion RT of each participant. The gray and black symbols represent the mean and individual values for each participant, respectively. The gray and black lines represent the mean and individual regression lines for each participant, respectively. (d) The mean values of light‐on RT of each participant. The gray and black symbols represent the mean and individual values for each participant, respectively. The gray and black lines represent the mean and individual regression lines for each participant, respectively.

Figure 4 shows the mean values (± standard deviation: SD) of the motion RT and light‐on RT for each eccentricity. Table 1 shows the mean and standard deviation of the motion RT and light‐on RT. The one‐way analysis of variance for the motion RT showed a main effect of eccentricity (*F* (4.000, 68.000) = 6.938, *p* < 0.001, Partial *η* ^*2*^ = 0.290). Post hoc multiple comparisons showed that motion RT at the eccentricity of 8°, 10°, and 12° was significantly longer than that at 4° (*p* < 0.05, *p* < 0.05, *p* < 0.001, respectively). The one‐way analysis of variance for the light‐on RT showed a main effect of eccentricity (*F* (2.748, 46.713) = 5.702, *p* < 0.05, Partial *η* ^*2*^ = 0.251). Subsequent post hoc multiple comparisons revealed a significant increase in light‐on RT at the eccentricity of 12° compared to 4 and 6° (*p* < 0.05 for both comparisons). These findings suggest a notable delay in motion RT at 8°, with no further change from 8° to 12°. In contrast, light‐on RT showed no change between 4° and 10°, but a significant delay at 12°.

**FIGURE 5:**
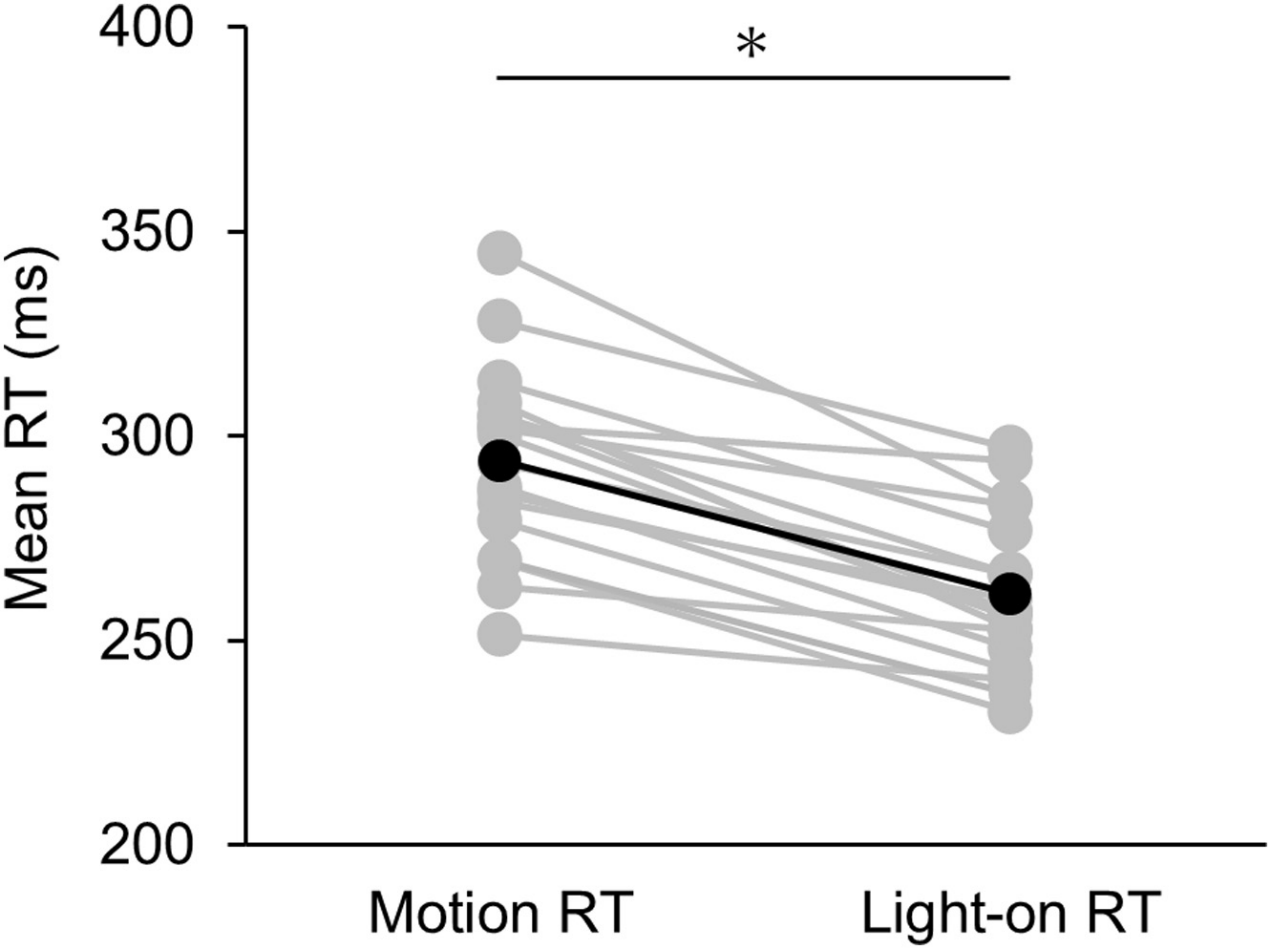
The mean reaction time including all eccentricity conditions for each participant in motion and light‐on RT. The gray and black symbols represent the mean and individual values for each participant, respectively. The asterisk indicates a significant difference in the mean values of motion and light‐on RT.

Figure 5 shows the mean and individual RTs for each participant, including all eccentricity conditions for the motion and light‐on RT tasks. We conducted a paired t‐test for each RT result and found a significant difference between the conditions (*t* (17) = 9.455, *p* < 0.001, *d* = 1.492). This result indicates that it is proved that motion RT and light‐on RT reflect different information processing processes. The cortical visual information processing includes two pathways: one for processing the color and shape of an object, and the other for processing the speed and direction of an object. In this study, the motion RT was a quick reaction to target motion which is primarily associated with the latter pathway, while the light‐on RT task did not involve target motion.

### 3.3 The correlation between TTC and RT

**FIGURE 6:**
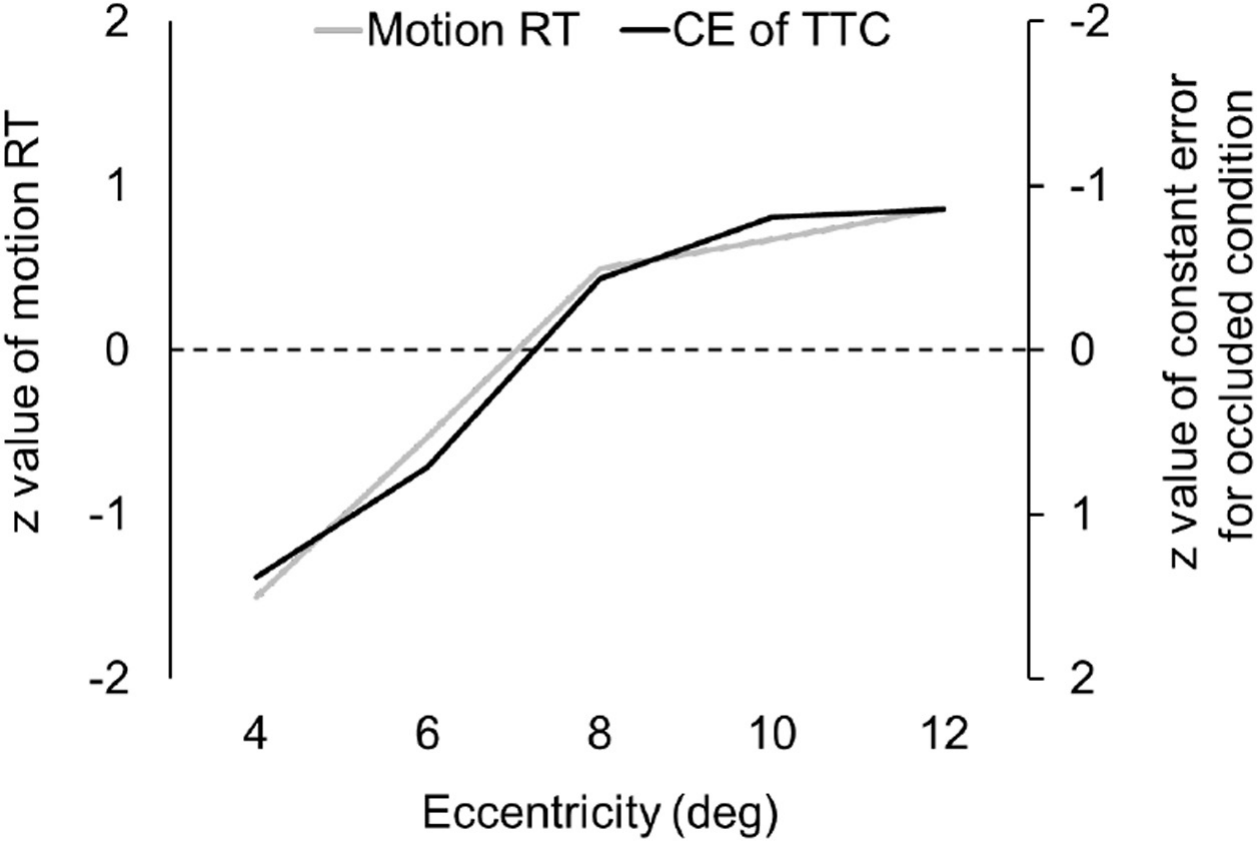
The relationship between the CE of TTC for the occluded condition and motion RT. The gray and black lines show *z* values of motion RT and CE of TTC for the occluded condition for each eccentricity, respectively. The *z* values of CE and axes are inverted for convenience.

Figure 6 shows the *z* value of motion RT and CE of TTC for the occluded condition of each eccentricity. For ease of comparison, CE shows the values of the axes inverted. Thus, Motion RT and CE are similarly affected by eccentricity, especially abruptly changed from 4° to 8°, and gradually changed from 8° to 12°.

## 4 DISCUSSION

The purpose of this study was to verify the effect of eccentricity on visual motion prediction based on speed perception in peripheral vision. We attempted to evaluate perceptual accuracy and speed to clarify the properties of peripheral visual function from various perspectives.

### 4.1 Effect of eccentricity on TTC error

Our results showed that the TTC error was affected by eccentricity in peripheral vision. The CE of the TTC task implies the bias of early or late responses with respect to the time the target coincided with the goal. In the present study, we used an occluded task to estimate coincidence anticipation timing. In this study, the CE of the TTC showed delayed responses at all eccentricities in both visible and occluded conditions using the speed of 8°/s. Previous studies have shown that TTC errors are affected by target speed, with higher speed resulting in a delayed response (Bennett et al., 2010; Miyamoto et al., 2021; Peterken et al., 1991). Miyamoto et al. (2021) have shown that the response is delayed from the actual coincidence time when the target velocity is 5°/s. Bennett and colleagues have also shown that a significant velocity effect was shown when the actual TTC was 1 s or more, and the delay response was shown at a relatively faster speed. Based on this finding, we set the target speed to 8°/s, which may have caused the overall delayed error. Our results showed that for the CE in the occluded condition, the response became earlier as the eccentricity increased. This result is consistent with our hypothesis and suggests that participants perceive slower target velocity as eccentricity increases. In fact, TTC estimation uses information on the visible interval before target disappearance (Battaglini et al., 2013), and previous studies have reported that TTC error is affected by target velocity (Bennett et al., 2010; Miyamoto et al., 2021; Peterken et al., 1991).

Bennett et al. (2010) have reported that there is an early response of TTC for slow‐moving targets compared to medium and fast‐moving targets in goal fixation conditions (Bennett et al., 2010). Miyamoto et al. (2021) have also reported object velocity effects, in which even a 1°/sec increase in target velocity causes larger delayed errors. Previous studies have shown that visual motion stimulus in the peripheral vision is perceived more slowly and increases the threshold for motion as a function of eccentricity (Hassan et al., 2016; Tynan & Sekuler, 1982). Furthermore, a number of previous studies have reported that low contrast target is perceived slower than high contrast (Battaglini et al., 2013; Gegenfurtner et al., 1999; Stone & Thompson, 1992). Taken together, it is suggested that as eccentricity increases, the target velocity is perceived more slowly, resulting in earlier responses in the TTC task. Previous studies examining the influence of eccentricity on perceived time have reported that peripheral vision perceives time earlier, similar to this study (Jovanovic & Mamassian, 2020). However, since their task was to mark the timing when visual events were presented, the visual motion velocity was not relevant. Therefore, it is possible that earlier responses in peripheral vision are influenced by factors other than accuracy for speed perception of moving objects.

Regarding the comparison of results between the visible and occluded conditions, the CE of the TTC in the visible condition may have little predictive component because the target was visible until the end, and thus there was little effect of eccentricity. In contrast, the CE of the TTC in the occluded condition showed that the response became significantly earlier as the eccentricity increased. These results, as shown in Figure 2d, indicate that the response generally becomes earlier regardless of whether the CE is close to zero or far from it. This suggests that the observed results are not necessarily indicative of improved accuracy due to eccentricity.

### 4.2 Effect of eccentricity on motion RT and light‐on RT

Our results showed that the motion and light‐on RTs were longer as eccentricity increased. Several studies have shown that light‐on RT, or premotor time, meaning the time between stimulus presentation and the onset of muscle activity, increases as a function of visual field eccentricity (Ando et al., 2001, 2002; Berlucchi et al., 1971; Osaka, 1976, 1978). However, the effect of eccentricity on motion RT has never been investigated. The motion RT is the time between the motion onset and the button press, while the light‐on RT means the time between the target appearance and the button press. These methods would be able to evaluate the processing speed of the CNS (central nervous system). Visual information processing is distinguished into the ventral and dorsal pathways (Goodale & Milner, 1992). The former identifies the quality of the object (e.g., color and shape), while the latter identifies the visual motion (e.g., direction and speed). Since motion RT involves target motion, while light‐on RT does not involve target motion, motion RT could rely more on the dorsal pathway in visual information processing. Previous studies have shown a significant correlation between the motion RT and initiation of pursuit eye movement (Miyamoto et al., 2020; Ono et al., 2019). It is known that smooth pursuit is induced by the slip of the retinal image, and the pursuit initiation is associated with activity in the MT and MST areas that are part of the dorsal pathway (Dursteler & Wurtz, 1988; Komatsu & Wurtz, 1988; Newsome et al., 1988; Ono & Mustari, 2016). From this knowledge, motion RT indirectly reflects MT and MST activity, and the delay in motion RT could be due to the reduced sensitivity of the retinal slip. On the other hand, it has also been reported that visual acuity and contrast sensitivity are significantly reduced as retinal eccentricity increases (Hassan et al., 2016; Jacobs, 1979; Mandelbaum & Sloan, 1947; Rovamo & Virsu, 1979; Wright & Johnston, 1983) Taken together, both motion RT and light‐on RT could be affected by eccentricity, regardless of whether visual motion is involved or not.

### 4.3 Relationship between TTC error and RT

In the present study, the CE of TTC for the occluded condition and motion RT showed similar changes with eccentricity (Figure 6). TTC is considered to be necessary to predict the velocity of the target motion from the retinal slip, while motion RT is a task that responds quickly to target motion. Thus, both tasks could be indirectly associated with visual motion processing in the activity of the cortical areas. A number of previous studies have shown that visual motion stimulus in the peripheral vision is perceived more slowly and increases the threshold for motion as a function of eccentricity (Hassan et al., 2016; Tynan & Sekuler, 1982). This was the case even though stimuli have equal contrast in the central and peripheral vision (Hassan et al., 2016). Thus, the effect of eccentricity on TTC error for the occluded condition cannot be explained only by changes in the perception of object quality, such as visual acuity and contrast perception. In particular, the change of motion processing could be due to the neuronal activity in the areas MT and MST of the dorsal pathway. These areas contain direction and speed selective cells and show an increase in firing rate to the preference (Ilg, 2008; Krekelberg & van Wezel, 2013; Maunsell & Van Essen, 1983). It has been reported that lesions in the MT cause impairments in speed perception (Orban et al., 1995), and microstimulation of MT neurons leads to a bias in speed judgments toward preferred speed (Liu & Newsome, 2005). Maunsell and Van Essen (1983) have reported a slightly positive linear relationship between the optimal speed of MT neurons and eccentricity from 0° to 20° using monkeys (Maunsell & Van Essen, 1983). Therefore, it is most likely that a slower speed perception in peripheral vision delays the perceived speed of motion onset and leads to an earlier response in the TTC task.

## 5 CONCLUSION

We attempted to clarify the properties of visual motion responses in peripheral vision across different eccentricities. Our results demonstrated that the estimation of time‐to‐contact (TTC) in the occluded condition showed an earlier response as the eccentricity increased. In addition, the motion and light‐on RTs tended to become longer as the eccentricity increased. These results suggest that motion prediction and speed of motion perception are altered with increasing eccentricity. In future studies, it is necessary to investigate the effects of perceived speed on the TTC by varying the target speed and eccentricity.
